# Sympathetic nervous system activity and reactivity in women with gestational diabetes mellitus

**DOI:** 10.14814/phy2.14504

**Published:** 2020-07-06

**Authors:** Laura M. Reyes, Rshmi Khurana, Charlotte W. Usselman, Stephen A. Busch, Rachel J. Skow, Normand G. Boulé, Margie H. Davenport, Craig D. Steinback

**Affiliations:** ^1^ Program for Pregnancy and Postpartum Health University of Alberta Edmonton AB Canada; ^2^ Faculty of Kinesiology, Sport, and Recreation University of Alberta Edmonton AB Canada; ^3^ Women and Children's Health Research Institute (WCHRI) University of Alberta Edmonton AB Canada; ^4^ Departments of Medicine and Obstetrics and Gynecology Faculty of Medicine & Dentistry University of Alberta Edmonton AB Canada; ^5^ Alberta Diabetes Institute University of Alberta Edmonton AB Canada

**Keywords:** gestational diabetes mellitus, MSNA, pregnancy

## Abstract

**Introduction:**

Gestational diabetes mellitus (GDM) is associated with vascular dysfunction. Sympathetic nervous system activity (SNA) is an important regulator of vascular function, and is influenced by glucose and insulin. The association between GDM and SNA (re)activity is unknown. We hypothesize that women with GDM would have increased SNA during baseline and during stress.

**Methods:**

Eighteen women with GDM and 18 normoglycemic pregnant women (controls) were recruited. Muscle SNA (MSNA; peroneal microneurography) was assessed at rest, during a cold pressor test (CPT) and during peripheral chemoreflex deactivation (hyperoxia). Spontaneous sympathetic baroreflex gain was quantified versus diastolic pressure at rest and during hyperoxia.

**Results:**

Age, gestational age (third trimester) and pre‐pregnancy body mass index and baseline MSNA was not different among the groups. Women with GDM had a similar increase in MSNA, but a greater pressor response to CPT compared to controls (% change in MAP 17 ± 7% vs. 9 ± 9%; *p* = .004). These data are consistent with a greater neurovascular transduction in GDM (% change in total peripheral resistance/% change in burst frequency [BF]: 15.9 ± 30.2 vs. −5.2 ± 16.4, *p* = .03). Interestingly, women with GDM had a greater reduction in MSNA during hyperoxia (% change in BF −30 ± 19% vs. −6 ± 17%; *p* = .01).

**Conclusion:**

Women diagnosed with GDM have similar basal SNA versus normoglycemic pregnant women, but greater neurovascular transduction, meaning a greater influence of the sympathetic nerve activity in these women. We also document evidence of chemoreceptor hyperactivity, which may influence SNA in women with GDM but not in controls.

## Graphical abstract

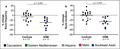

## 1 INTRODUCTION

Maternal glucose metabolism is altered during pregnancy to meet the demands of the growing fetus; as such, pregnancy is associated with progressive insulin resistance beginning in the second trimester (Catalano, Tyzbir, Roman, Amini, & Sims, 1991). In a subset of pregnancies, this adaptation uncovers metabolic abnormalities, which present as gestational diabetes mellitus (GDM). GDM is the most common pregnancy complication, affecting up to 17% of the obstetric population (Hunt & Schuller, 2007). It is defined as diabetes diagnosed in the second or third trimester of pregnancy in the absence of preexisting type 1 or type 2 diabetes (Diabetes Canada Clinical Practice Guidelines Expert Committee et al., 2018). Women with GDM have a twofold higher risk of cardiovascular events in the first decade after delivery (Kramer, Campbell, & Retnakaran, 2019). Yet, the fundamental link(s) between GDM and these health outcomes is unclear. Sympathetic hyperactivity is directly linked to cardiovascular and metabolic diseases in a number of clinical populations, including obesity, metabolic syndrome, and type 2 diabetes (Grassi, 2006; Malpas, 2010; Thorp & Schlaich, 2015). In nonpregnant populations, insulin resistance, hyperglycemia, and hyperinsulinemia have been identified as contributing factors leading to sympathetic hyperactivity (Anderson, Hoffman, Balon, Sinkey, & Mark, 1991; Berne, Fagius, Pollare, & Hjemdahl, 1992; Rowe et al., 1981). During pregnancy, Shi et al. (2019) showed that insulin transport across the blood‐brain barrier and brain insulin degradation are increased. However, pregnancy was associated with central resistance to insulin and leptin.

The peripheral chemoreflex (the carotid bodies) is an important regulator of sympathetic activity. In humans, radiological evidence indicates that the carotid bodies are enlarged in people with diabetes, suggestive of hyperplasia and hyperactivity (Cramer et al., 2014). We have recently shown that the chemoreflex is a contributor to sympathetic activation following glucose ingestion in non‐pregnant individuals (Smorschok et al., 2018). Furthermore, ground‐breaking work by Ribeiro et al. (2013) in a rodent model of diabetes demonstrated that surgical removal of the carotid bodies not only reduced sympathetic nervous activity (SNA), but also prevented insulin resistance as well as subsequent hypertension. These data suggest that hyperactivity of the chemoreflex may be associated with the pathophysiology of the short and long‐term cardiovascular consequences of diabetes in nonpregnant populations with diabetes. Whether heightened chemoreflex activity contributes to the pathology of GDM remains to be established.

We hypothesize that women with GDM would have increased sympathetic activity during baseline and during stress. Thus, we conducted the first assessments of sympathetic nervous system function in GDM, including evaluating the carotid bodies as a primary mechanism of control.

## 2 METHODS

All procedures contributing to this work comply with the ethical standards set by the latest revision of the Declaration of Helsinki and had been approved by the Health Research Ethics Board at the University of Alberta (Pro00041144). Written informed consent was obtained from all study participants before testing.

### 2.1 Participants

Eighteen normotensive women with singleton pregnancies diagnosed with GDM who were nonsmokers, and free of cardiovascular, respiratory or neurological diseases were recruited at the Royal Alexandra Hospital or the Gestational Diabetes Clinic in Edmonton, Alberta, Canada. In addition, 18 normotensive, euglycemic women with singleton pregnancies were recruited at the Program for Pregnancy and Postpartum Health. There were no differences in mean age, gestational age and pre pregnancy body mass index (BMI) between groups. Descriptive data, including basal sympathetic activity, from the normotensive, euglycemic women with singleton pregnancies has been already published (Charkoudian et al., 2017). However, this study focuses on the novel assessment of women with GDM.

### 2.2 Experimental design

Normotensive euglycemic pregnant women (controls) arrived at the Program for Pregnancy and Postpartum Health at 9:00 a.m. after an overnight fast (12 hr), where a peripheral intravenous catheter was placed in the participant's left arm, blood samples were drawn and a standardized light meal was provided (toasted bagel with sugar‐free jam and juice box). For the women with GDM recruited at the Royal Alexandra Hospital (*n* = 8), the test was performed at the hospital in a designated research space (Lois Hole Hospital Women's Research Centre). Blood samples for the women with GDM were performed in either fasted (*n* = 6) or not fasted state (*n* = 12). All of the participants reported avoiding caffeine and strenuous exercise 12‐hr prior to testing. No instructions regarding avoiding medications prior to the test were given to the participants. All the tests were done between 60 and 120 min following food ingestion.

Heart rate was measured continuously using a standard electrocardiogram (lead II). Blood pressure was measured continuously using finger photoplethysmography (Finometer Pro; Finapres Medical Systems) and mean, systolic, and diastolic pressures (MAP, SBP, and DBP, respectively) were identified from the pressure waveform. Participants were seated in a semi‐recumbent position in a darkened room. Cardiac output was assessed using the Modelflow algorithm from the Finometer blood pressure waveform (Wesseling, Jansen, Settels, & Schreuder, 1985). Beat‐by‐beat total peripheral resistance (TPR) was calculated as: MAP/cardiac output.

Muscle SNA (MSNA) was recorded via peroneal microneurography as previously described (Usselman et al., 1985). Briefly, a tungsten microelectrode (35–60 mm in length, 200 μm in diameter) was inserted transcutaneously into the common peroneal nerve dorsal to the head of the fibula. MSNA was confirmed by the occurrence of pulse‐synchronous bursts of activity and an increased firing frequency during voluntary apnea and an absence of a response to a loud noise (Usselman et al., 1985). A 10 min baseline was recorded followed by a cold pressor test (CPT) and a hyperoxia protocol.

### 2.3 Cold pressor test and hyperoxia protocols

For both protocols, a 3‐min baseline was recorded. Hemodynamic measurements as well as MSNA were acquired continuously. During the CPT, participants put their hand in an ice bath (~0 to 4°C) up to the wrist for 3 min. A heating pad was used to subsequently rewarm their hand following the test. During the hyperoxia protocol, participants breathed 100% oxygen for 3 min through a standard oro‐nasal face mask for comfort. The order of these protocols was randomly allocated, and separated by sufficient time for recovery of blood pressure, heart rate, and MSNA to resting values (approximately 10 min).

### 2.4 Muscle sympathetic activity data analysis

We used a semi‐automated algorithm (ADInstruments Peak Detection) to identify bursts of sympathetic activity. These were then confirmed by a trained observer (CDS/LMR). Sympathetic activation was quantified as burst frequency (bursts/min; BF) and burst incidence (bursts/100 heart beats; BI). We also analyzed the amplitude of each burst at rest and during each protocol as previously described (Usselman et al., 2015). Briefly, the largest burst amplitude for each participant at baseline was assigned used to normalize burst amplitude occurring during the CPT and the hyperoxia protocols. The median amplitude was used to compare responses between the groups.

Each protocol was analyzed in 1‐min bins. The bin with the greatest hemodynamic response and change in MSNA was defined as the peak (i.e., during CPT) or nadir (i.e., during hyperoxia) response. We calculated the absolute and percentage change of MAP, SBP, DBP, BF, and BI between baseline and peak/nadir for each protocol. Neurovascular transduction was calculated as the percentage change in TPR by the percentage change in MSNA (BF, BI; Usselman et al., 2015).

Similar to previous work, custom action potential detection software (APD v2.1; Salmanpour, Brown, & Shoemaker, 2010) was used to determine the pattern of recruitment of neurons during acute periods of sympathetic stress (Schmidt et al., 2018). Briefly, 100 consecutive sympathetic bursts from each participant were exported, and extracellular sympathetic action potentials were identified and counted. Action potential amplitudes were normalized to the largest sympathetic action potential within the data. Action potentials were “clustered” based on amplitude into groups approximating single or small groups of sympathetic neurons (Schmidt et al., 2018). Action potential data were expressed as average number of action potentials per burst, average number of “clusters” per burst and total number of action potentials per minute.

Spontaneous sympathetic baroreflex gain was evaluated at rest (10 min) and during hyperoxia (entire 3‐min). MSNA data were shifted backward so that the peak of each sympathetic burst coincided with the diastolic period, which initiated it. Diastolic blood pressure data were then arranged into 2‐mmHg bins. The percent occurrence of a sympathetic burst (ranging from 0% to 100%) within each DBP bin provided the values of sympathetic BI (Usselman et al., 1985). The slope of the relationship between BI and DBP was taken to represent sympathetic baroreflex gain as described previously (Usselman et al., 1985).

### 2.5 Blood analyses

Blood samples were centrifuged, separated and store at −80°C until analysis. Baseline serum glucose (hexokinase, Seimens Advia 1800), insulin (chemiluminescence microparticle immunoassay, Abbott Architect i2000), estradiol (electrochemiluniscence, Roche Cobas) progesterone (chemilunimescence competitive immunoassay, Siemens Centaur) and testosterone (two‐site sandwich chemiluninescence, Siemens Centaur) were assessed by a commercial laboratory (DynaLIFE).

Plasma adrenaline, noradrenaline, and neuropeptide Y (NPY) concentrations were assessed at baseline. In addition, plasma was also collected during each protocol's baseline and during minute 2–3 of the CPT and hyperoxia protocol. Adrenaline and noradrenaline were determined according to manufacturer's instructions using a competitive ELISA kit (BA E‐5400, LDN), while NPY was determined using a sandwich ELISA (LA‐F12183, LSBio, LifeSpan BioSciences, Inc.). The inter‐ and intra‐assay coefficients of variation obtained were: adrenaline 6.1% and 15%, respectively. Noradrenaline 4% and 5%, respectively, and NPY: 4.4% and 3.8%, respectively.

### 2.6 Statistical analysis

The Shapiro–Wilk test was used to assess the normality of the continuous data. Descriptive data were expressed as mean and standard deviation (*SD*). Groups were compared using unpaired *t* test or Mann–Whitney test when appropriate. Categorical variables were analyzed by chi‐square (gestational weight gain) or Fisher's exact test (ethnicity). Outliers were removed using a Grubbs' test. Differences in action potential firing were analyzed using an ANCOVA controlling for signal‐to‐noise ratio and R–R interval. Statistical significance was defined a priori as *p* < .05. Using the difference between two independent means (% change in BF in hyperoxia as a primary outcome); assuming two tails; a power of 0.8 and an alpha of 0.05 a sample size of 10 in each group was computed (G*Power 3.1.9.2; Software). We recruited 18 women in each group to account for the MSNA data that we were not able to acquire or the data that were lost during the protocol. Thus we have the following number of participants per condition: *at baseline:* 18 controls and 12 women with GDM; *during CPT:* 18 controls and 11 women with GDM; and *during hyperoxia:* 17 controls and 11 women with GDM. Data were analyzed using GraphPad Prism 7 statistical software (GraphPad Software) or SigmaPlot^TM^ (ANCOVA analysis [Systat Software]).

## 3 RESULTS

### 3.1 Baseline characteristics

**FIGURE 1:**
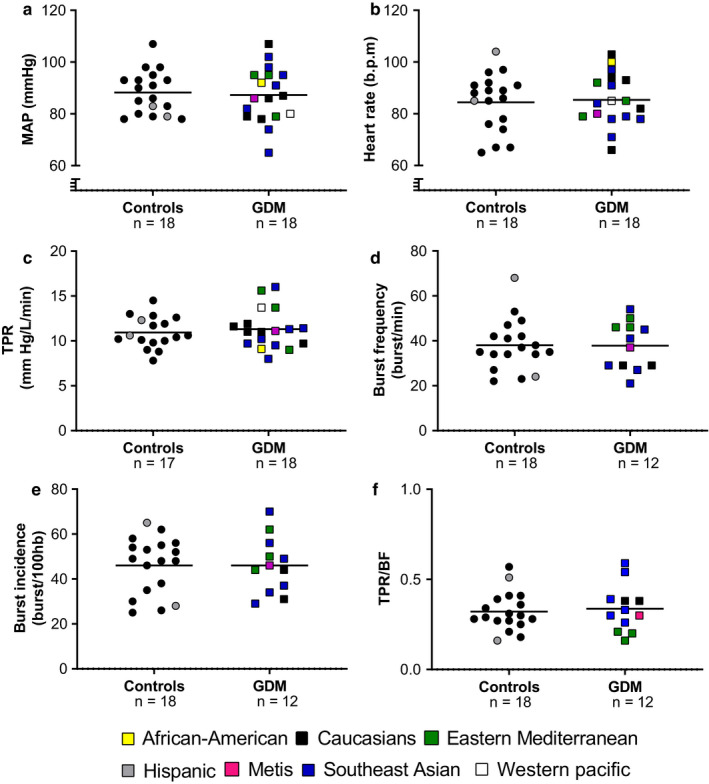
Baseline hemodynamics and muscle sympathetic nervous activity in normotensive, euglycemic pregnant women (controls) and women with gestational diabetes mellitus (GDM). (a) Mean arterial blood pressure (MAP); (b) heart rate; (c) total peripheral resistance (TPR); (d) burst frequency (BF); (e) burst incidence; neurovascular transduction (f; TPR/BF) in normotensive euglycemic pregnant women (controls, circles, *n* = 17–18) and normotensive women with GDM (GDM; squares, *n* = 12–18). Ethnicity is represented by colors as following: African–American = yellow; Caucasians = black; Eastern Mediterranean = green; Hispanic = grey; Metis = magenta; Southeast Asian = blue and Western pacific (Asia) = white. Groups were compared using unpaired *t* test or Mann–Whitney test when appropriate

Age, gestational age, and anthropometric characteristics (pre‐pregnancy BMI, current weight, BMI, and gestational weight gain) were similar between groups. There were no differences among the groups regarding any hemodynamic parameter at baseline. (Table 1; Figure 1a–c).

**TABLE 1:** Participant's baseline characteristics

	Controls (*n* = 18)	Women with GDM (*n* = 18)	*p*‐value
General characteristics			
Age (years)	30 ± 4	33 ± 4	.1
Weeks of gestation	32 ± 5	33 ± 3	.9
Weight (kg)	80 ± 14	84 ± 17	.4
Height (m)	1.7 ± 0.1	1.6 ± 0.1	.05
BMI (kg/m^2^)	28.4 ± 4.8	31.4 ± 5.6	.06
Pre‐Preg BMI (kg/m^2^)^a^	25.1 ± 5.2	27.1 ± 5.7	.07
Ethnicity (*n*)			**.001**
African‐American	0	1	
Caucasian	16	5	
Eastern Mediterranean	0	3	
Hispanic	2	0	
Metis	0	1	
Southeast Asian	0	7	
Western Pacific	0	1	
Gestational weight gain category (%)^b^			.3
Inadequate	11	31	
Normal	56	31	
Excessive	33	38	
Medications (number of participants)			
Insulin	0	6	
Diclectin	0	1	
Synthroid	0	1	
Metformin	1	1	
Baseline hemodynamics			
Systolic blood pressure (mmHg)	115 ± 11	114 ± 13	.8
Diastolic blood pressure (mmHg)	71 ± 7	70 ± 10	.8
Cardiac output (L/min)^c^	8 ± 2	8 ± 2	.9
Metabolic and hormone status^d^			
Glucose (mmol/L)	4.1 ± 0.3	5.7 ± 2.4	**.001**
Insulin (mmol/L)	52.1 ± 25.1	227 ± 213.3	**<.0001**
Estradiol (pmol/L)	59,168 ± 18,410	78,155 ± 55,845	.2
Progesterone (nmol/L)	514.2 ± 244.4	490.3 ± 197.3	.8
Testosterone (nmol/L)	2.3 ± 0.7	3.6 ± 2	**.03**

Bold values: *p* < .05.

Abbreviations: BMI, body mass index; GDM, gestational diabetes mellitus.

^a^ Pre‐pregnancy BMI was calculated using self‐reported pre‐pregnancy weight.

^b^ Gestational weight gain category was determined using the Guidelines for weight gain during pregnancy (Institute of Medicine (US) and National Research Council (US) Committee to Reexamine IOM Pregnancy Weight Guidelines, 2009).

^c^ Cardiac output and total peripheral resistance were calculated using the ModelFlow algorithm (Wesseling et al., 1985).

^d^ Blood samples were not collected in the fasted state in 12 women with GDM. Groups were compared using unpaired *t* test or Mann–Whitney test when appropriate. Categorical variables were analyzed by chi‐square (gestational weight gain) or Fisher's exact test (ethnicity). Data expressed as mean ± *SD*.

Glucose and insulin were as expected (non‐fasted) higher in women with GDM compared to controls. Interestingly, testosterone concentrations were higher in women with GDM compared to controls (Table 1). Basal sympathetic activity (BF, BI and burst amplitude, total activity) was similar between groups (Figure 1d–f; Table 2). The multi‐unit analysis indicated that the average number of active neurons within a given burst, the total pool of active neurons (clusters) and the firing frequency of active neurons were also not different between the groups (Table 2). Anthropometric characteristics, baseline hemodynamics, metabolic and hormone status in women with GDM that had MSNA measurements are presented in Table S1.

**TABLE 2:** Resting sympathetic function

	Controls (*n* = 18)	Women with GDM (*n* = 12)	*p*‐value
Burst amplitude (% of max amplitude)	49 ± 4	39 ± 14	.1
Total sympathetic activity (% max amplitude/min)	1,790 ± 495	1567 ± 742	.3
Neurovascular transduction (TPR/BF) × 100	32 ± 10	34 ± 13	.7
Sympathetic baroreflex gain			
Slope (bursts/100 hb/mmHg)	−3.3 ± 1.7	−4 ± 1.5	.2
Intercept (bursts/100 hb)	327 ± 106	268 ± 128	.2
Action potentials within a given burst	7 ± 3	8 ± 7	.6
Action potentials per minute	206 ± 144	212 ± 198	.9
Total pool of clusters (active neurons)	17 ± 5	16 ± 8	.9
Clusters per burst	3 ± 1	4 ± 3	.4

Abbreviations: BF, burst frequency; GDM, gestational diabetes mellitus; TPR, total peripheral resistance.

At baseline, there were no differences among the groups regarding their adrenaline concentrations (37.1 ± 9.6 pg/ml in controls vs. 37.6 ± 9.8 pg/ml in women with GDM), noradrenaline concentrations (337.6 ± 136.2 in controls vs. 340.6 ± 93.8 pg/ml in women with GDM), and NPY concentrations (992 ± 368 in controls vs. 1,101 ± 456 pg/ml in women with GDM). Resting sympathetic baroreflex gain and set‐point (intercept) were similar in GDM and control women indicating similar basal blood pressure control (Table 2).

None of the above‐mentioned parameters were different between the women with GDM recruited in the Program for Pregnancy and Postpartum Health laboratory and the women with GDM recruited in the Royal Alexandra Hospital.

### 3.2 Cold pressor test

**FIGURE 2:**
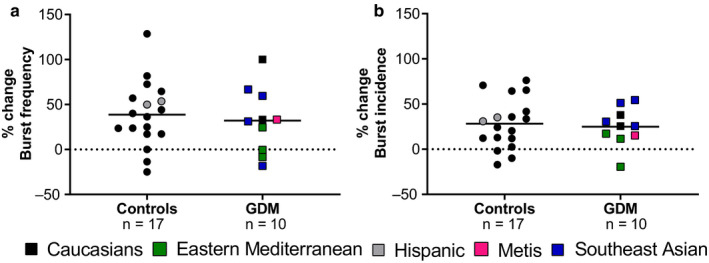
Changes in muscle sympathetic nervous activity during a cold pressor test in normotensive, euglycemic pregnant women (controls) and women with gestational diabetes mellitus (GDM). Percentage change in (a) burst frequency and (b) burst incidence during a cold pressor test in normotensive euglycemic pregnant women (controls, *n* = 17) and normotensive women with GDM (GDM; *n* = 9–10). Ethnicity is represented by colors as following: Caucasians = black; Eastern Mediterranean = green; Hispanic = grey; Metis = magenta and Southeast Asian = blue. Groups were compared using unpaired *t* test or Mann–Whitney test when appropriate

The cold pressor test elicited as similar increase in BF (*p* = .5; Figure 2a) and BI (*p* = .7; Figure 2b) in women with GDM and controls. There was also no difference in the number of sympathetic neurons recruited. This was mirrored by the catecholamines response (adrenaline: Δ 22.8 ± 30.3 in controls vs. 10.3 ± 1.4 pg/ml in women with GDM, *p* = .5; noradrenaline Δ 82.8 ± 154.7 in controls vs. 76.6 ± 63.1 pg/ml in women with GDM, *p* = .9). As well as the NPY response (Δ −6.8 ± 159.1 in controls vs. 50.3 ± 17.2 pg/ml in women with GDM, *p* = .9).

**FIGURE 3:**
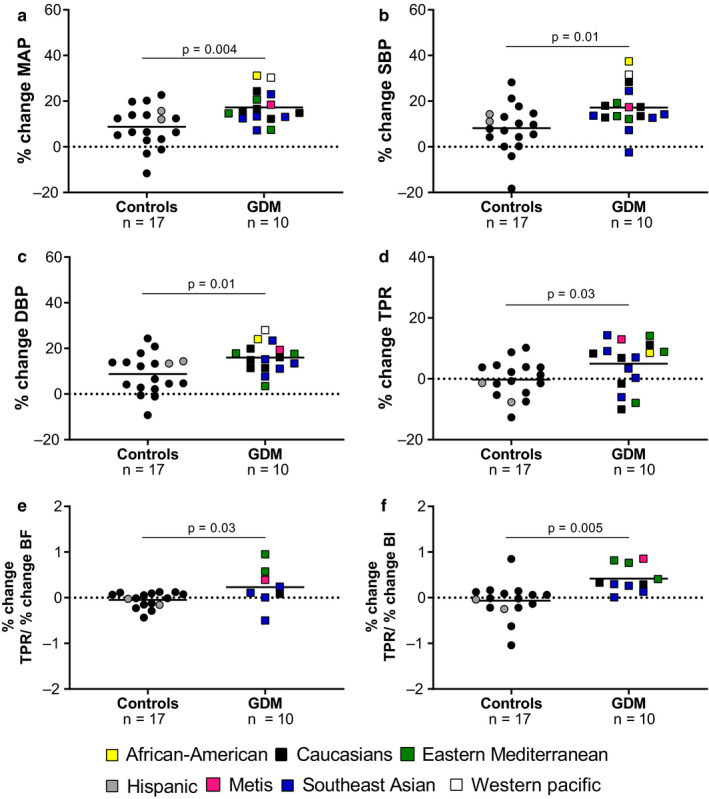
Changes in blood pressure and neurovascular transduction during a cold pressor test in normotensive, euglycemic pregnant women (controls) and women with gestational diabetes mellitus (GDM). Percentage change in (a) mean arterial blood pressure (MAP); (b) systolic blood pressure (SBP); (c) diastolic blood pressure (DBP); (d) total peripheral resistance (TPR); neurovascular transduction (e; percentage change in TPR/percentage change in BF); neurovascular transduction (f; percentage change in TPR/percentage change in BI) during a cold pressor test in normotensive euglycemic pregnant women (controls, *n* = 16–18) and normotensive women with gestational diabetes (GDM; *n* = 9–17). Ethnicity is represented by colors as following: African–American = yellow; Caucasians = black; Eastern Mediterranean = green; Hispanic = grey; Metis = magenta; Southeast Asian = blue and Western pacific (Asia) = white. Groups were compared using unpaired *t* test or Mann–Whitney test when appropriate

Despite a similar increase in sympathetic outflow during the CPT, the peak (%) increase in MAP, SBP, DBP, and TPR was greater in women with GDM compared to controls (Figure 3a–d). Thus, women with GDM had a higher neurovascular transduction during sympathetic activation compared to controls (Figure 3e,f).

Response to the cold pressor test was similar between the women with GDM recruited in the Program for Pregnancy and Postpartum Health laboratory and the women with GDM recruited in the Royal Alexandra Hospital.

### 3.3 Hyperoxia response

**FIGURE 4:**
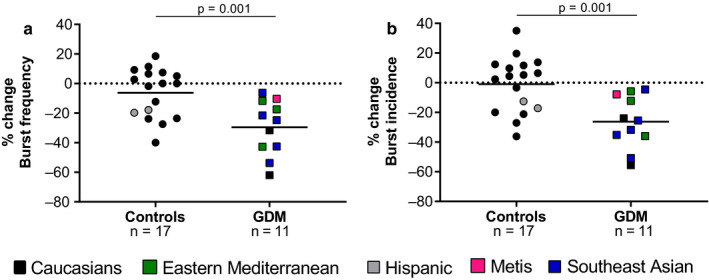
Changes in muscle sympathetic nervous activity during hyperoxia in normotensive, euglycemic pregnant women (controls) and women with gestational diabetes mellitus (GDM). Percentage change in (a) burst frequency and (b) burst incidence during hyperoxia in normotensive euglycemic pregnant women (controls, *n* = 17) and normotensive women with gestational diabetes (GDM; *n* = 11). Ethnicity is represented by colors as following: Caucasians = black; Eastern Mediterranean = green; Hispanic = grey; Metis = magenta and Southeast Asian = blue. Groups were compared using unpaired *t* test or Mann–Whitney test when appropriate

Exposure to hyperoxia significantly decreased BF and BI in women with GDM (Figure 4a,b). This attenuation was specific to the occurrence of bursts, as amplitude and the number of active neurons remained the same in each group (data not shown). There was a similar effect on hemodynamic variables in both groups: % change in MAP −1.6 ± 2.9% in controls versus −2.7 ± 3.1% in women with GDM, *p* = .3; % change in SBP: −2.1 ± 3.3% in controls versus −1.9 ± 2.8% in women with GDM, *p* = .8; % change in DBP −1.4 ± 2.8% in controls versus −2.1 ± 2.8% in women with GDM, *p* = .4.

Regarding catecholamine responses during hyperoxia, we found changes in adrenaline (Δ 0.1 ± 12.5 in controls vs. Δ −10.3 ± 9.2 in women with GDM; *p* = .07); and noradrenaline (Δ 48.3 ± 84.7 in controls vs. Δ 37.6 ± 128.9 pg/ml in women with GDM; *p* = .8) were not different between the groups. Similarly, NPY responses during hyperoxia were not different between groups (Δ 21.1 ± 125.7 pg/ml in controls vs. Δ 23.9 ± 109.0 pg/ml in women with GDM; *p* = .9).

Finally, hyperoxia did not change baroreflex gain (−3.8 ± 2.62 in controls vs. −4 ± 2.8 burst/100 heart‐beats/mmHg in women with GDM; *p* = .8) or set‐point (327 ± 197.1 in controls vs. 302.5 ± 165.6 a.u. in women with GDM; *p* = .7) in either group.

Response to hyperoxia was similar between the women with GDM recruited in the Program for Pregnancy and Postpartum Health laboratory and the women with GDM recruited in the Royal Alexandra Hospital.

## 4 DISCUSSION

To the best of our knowledge, this is the first assessment of sympathetic nervous system function (activity and reactivity to stress) in women with GDM including assessments of mechanisms of control (chemoreflex, baroreflex, and response to hyperoxia). Although MSNA was similar between groups at rest, women with GDM had an augmented blood pressure response to CPT consistent with a greater sympathetic neurovascular transduction. Furthermore, we demonstrated a reduction in sympathetic activity during hyperoxia in women with GDM but not normoglycemic women, suggesting the augmented chemoreflex activity in women with GDM.

Previous studies examining autonomic function in women with GDM have shown that women with GDM can have either no changes in heart rate variability (Heiskanen et al., 2010; Maser, Lenhard, & Kolm, 2014), or lower parasympathetic cardiac tone at rest (Poyhonen‐Alho et al., 2010; Weissman, Lowenstein, Peleg, Thaler, & Zimmer, 2006). In addition, Poyhonen‐Alho et al. (2010) found that plasma noradrenaline concentrations were similar in women with GDM and euglycemic pregnant controls (Figure 4).

The above‐mentioned data as well as ours suggest that women with GDM do not have a measurable increase in sympathetic tone at rest, which differs from previous data on women with hypertensive disorders of pregnancy (Fischer et al., 2004; Greenwood, Scott, Stoker, Walker, & Mary, 2001; Greenwood, Scott, Walker, Stoker, & Mary, 2003; Greenwood, Stoker, Walker, & Mary, 1998; Schobel, Fischer, Heuszer, Geiger, & Schmieder, 1996). However, our data demonstrate that women with GDM had a higher blood pressor response (higher neurovascular transduction) under stress (cold pressor test). This is important to consider, since increased pressor responses have been associated with increased risk of developing cardiovascular diseases later in life (Esler, Lambert, & Jennings, 1990; Grassi et al., 2007).

We did not observe any effect of GDM on baroreflex gain in our participants (both at rest and during hyperoxia). This is in keeping with previous data derived from head‐up tilt, Valsalva ratio, expiration/inspiration ratio, where it has been determined that women with GDM had similar autonomic responses compared to controls (Heiskanen et al., 2010; Maser et al., 2014; Weissman et al., 2006). Our data contribute to this understanding through the direct assessment of sympathetic regulation of blood pressure.

There are different potential mechanisms leading to sympathoexcitation during a normotensive pregnancy. Peripherally: estradiol and progesterone have been proposed as potential mechanisms contributing to sympathoexcitation (Reyes, Usselman, Davenport, & Steinback, 2018). Centrally: Pregnancy is known to alter the paraventricular nucleus (PVN) and arcuate nucleus in the hypothalamus to increase sympathetic tone. Specifically, it is known that during pregnancy the activity of NPY neurons, which inhibit the PVN presympathetic neurons, decreases, while the pro‐opiomelanocorticotropin neurons activity increases (Shi, Cassaglia, Gotthardt, & Brooks, 2015). Recent data from Shi et al. (2019) showed that pregnancy was associated with central resistance to insulin and leptin. Hence, since women with GDM have a greater insulin resistance compared to women with normoglycemic pregnancies, we believe that in women with GDM these mechanisms occur in parallel with an increase in chemoreceptor activity/sensitivity.

Chemoreflex sensitization is a contributing factor to neurovascular dysfunction in many clinical disorders, including heart and renal failure, hypertension and sleep apnea (Kara, Narkiewicz, & Somers, 2003; Prabhakar & Peng, 1985). Evidence have suggested that both glucose (Koyama et al., 2000; Smorschok et al., 2018) and insulin (Limberg, Curry, Prabhakar, & Joyner, 2014) levels play a role in the sensitivity of the chemoreflex during acute and chronic stimulation. For instance, Smorschok et al. (2018) found that in non‐pregnant populations, the SNA response to glucose ingestion (75 g glucose drink) is blunted (decrease of approximately 35%) with hyperoxia. It is important to acknowledge that postprandial glucose/insulin could have remained higher in women with GDM, and therefore, hyperoxia would be more effective in blunting SNA in this population. It has been shown that hyperoxia decreases SNA in people with heart transplant (Ciarka et al., 2005), renal failure (Hering et al., 2007), chronic obstructive pulmonary disease (Heindl, Lehnert, Criee, Hasenfuss, & Andreas, 2001), and obstructive sleep apnea (Narkiewicz et al., 1998). Our data suggest that desensitization of the chemoreflex by hyperoxia could be a novel line of inquiry related to the development and treatment of cardiovascular dysfunction associated with GDM. Our data support that hyperactivity of the chemoreflex may contribute to a greater support of resting activity and more pronounced SNA reactivity to hyperoxia in women with GDM.

The changes in blood pressure following hyperoxia were minimal and not statistically significant among the groups. In conjunction with a significant decrease in SNA, these findings suggest that neurovascular transduction may differ during sympathetic activation versus deactivation, as observed previously in normotensive normoglycemic women (Steinback et al., 2019).

We did not find any differences in the catecholamines concentrations in our populations both at rest and under stress. Since under stress, women with GDM had a higher pressor response and a greater transduction of SNA to the vasculature we could speculate that women with GDM may have an increase in the sensitivity or number of the vascular catecholamines receptors and thus, their response was greater in comparison to normotensive, euglycemic pregnant women (Davidge & McLaughlin, 1992). More research is needed to determine the number and sensitivity of the catecholamines receptors in the vascular wall of these women.

One of the strengths of our experimental approach is that age, gestational age, gestational weight gain and pre‐pregnancy BMI, which are variables that influence SNA were no different between the groups. Moreover there were no differences among the groups regarding gestational weight gain. Therefore, differences between the groups regarding sympathetic activity are not explained by these factors. Moreover this is the first study that has provided an extended evaluation of neurovascular communication by directly assessing sympathetic activity during rest and during stress using microneurography and surrogate markers of sympathetic activity such as plasma levels of catecholamines.

Our study has some limitations; some women with GDM were recruited upon admission to the hospital, and their glucose levels may not have been controlled. In addition, not all of the blood samples at rest from the women with GDM were taken fasted. Nonetheless, this would not affect our hyperoxia results since the participants that arrived fasted to the test were provided with a light meal after the blood draw. Moreover, all the women recruited completed their test within 60–120 min of their meal, hence, all the reflex testing was performed under the same conditions between the groups. Finally, we analyzed MSNA and hemodynamics during the three different baselines (rest [3 min]), baseline for the cold pressor test (3 min) and the baseline for the hyperoxia challenge (3 min; data not shown) and found that they were not different among women with GDM suggesting that insulin and glucose concentrations did not affect our results.

Women with GDM in our group came from different ethnic backgrounds. In particular, we had more women from Southeast Asian and Middle‐Eastern descent in our GDM group and more Caucasian women in our control group. To our knowledge, comparisons of MSNA between South Asian and Caucasian populations (pregnant or otherwise) have not been conducted. A study by Okada et al. (2015) found that pregnant East‐Asian women have lower baseline MSNA, lower noradrenaline concentrations, higher aldosterone and estradiol concentrations compared to Caucasian pregnant women. In addition, MSNA and blood pressure response to stress (tilt‐table test) was lower compared to controls. We acknowledge it is possible that differences in ethnicity may influence our results. However, the distribution and variability of basal MSNA and MSNA reactivity were similar between groups, and importantly, the distribution of data from Caucasian women with GDM appeared no different from women with GDM from other ethnicities. Furthermore, Okada et al. concluded that the main contributing to lower MSNA in their cohort of East‐Asian pregnant women were a lower pre‐pregnancy BMI and higher levels of estradiol. We did not observe any difference in estradiol between our two groups of women. Furthermore, our current cohort of women were matched for pre‐pregnancy BMI. Thus, while we believe that the role of ethnicity in MSNA in our data is minimal, there is clearly a gap within the broader literature with respect to characterizing sympathetic regulation in diverse ethnic groups.

In conclusion, we presented the first assessments of sympathetic nervous system function in women with GDM, focusing on multiple mechanisms of control. We found that women with GDM have an increased pressor response under stress, likely due to elevated neurovascular transduction. Our data suggest that the chemoreflex plays a greater role in support of baseline MSNA in women with GDM. Potentially, this could lead to the development of cardiovascular diseases later in life in these women.

## CONFLICTS OF INTEREST

The authors report no conflicts of interest and that the funding sources did not play a role in the design, collection, analysis and interpretation of the data, in the writing of the manuscript and the decision to submit the manuscript for publication.

## AUTHOR CONTRIBUTIONS

CDS, MHD, RK, and NGB contributed to concept and design of the study. LR, CWU, SAB, and RJS contributed to data collection and analysis. LR contributed to first draft of the article. All authors contributed to critical revision and approval of the manuscript.

## Supporting information



Table S1
