# Supplementary material for: Sympathetic nervous system activity and reactivity in women with gestational diabetes mellitus
Source: Physiol Rep. 2020 Jul 6;8(13):e14504. doi: 10.14814/phy2.14504 (PMC7338594; doi:10.14814/phy2.14504)
Supplement: Supplementary file 1 — Table S1 [file PHY2-8-e14504-s001.docx]

**Supplementary table. Participant’s baseline characteristics**

|  | **Controls** | **Women with GDM** | **p-value** |
| --- | --- | --- | --- |
|  | **(n=18)** | **(n=12)** |  |
| *General characteristics* |  |  |  |
| Age (years) | 30 ± 4 | 33 ± 4 | 0.1 |
| Weeks of gestation | 32 ± 5 | 33 ± 3 | 0.6 |
| Weight (kg) | 80 ± 14 | 79 ± 12 | 0.9 |
| Height (m) | 1.7 ± 0.1 | 1.6 ± 0.01 | **0.01** |
| BMI (kg/m^2^) | 28.4 ± 4.8 | 30.2 ± 4.3 | 0.2 |
| Pre-Preg BMI (kg/m^2^)^*^ | 25.1 ± 5.2 | 26.5 ± 4.3 | 0.5 |
| Ethnicity (n) ^†^ |  |  | **0.001** |
| Caucasian | 16 | 2 | . |
| Eastern Mediterranean | 0 | 3 | . |
| Hispanic | 2 | 0 | . |
| Metis | 0 | 1 | . |
| Southeast Asian | 0 | 6 | . |
| Gestational weight gain category (%)^†^ |  |  | 0.9 |
| Inadequate | 11 | 30 |  |
| Normal | 56 | 40 |  |
| Excessive | 33 | 30 |  |
| Medications (number of participants) |  |  |  |
| Insulin | 0 | 5 | . |
| Synthroid | 0 | 1 | . |
| Metformin | 1 | 1 | . |
|  |  |  |  |
| *Baseline Hemodynamics* |  |  |  |
| Systolic blood pressure (mmHg) | 115 ± 11 | 114 ± 14 | 0.9 |
| Diastolic blood pressure (mmHg) | 71 ± 7 | 71 ± 7 | 0.7 |
| Mean arterial blood pressure (mmHg) | 88 ± 8 | 89 ± 12 | 0.9 |
| Heart rate (bpm) | 84 ± 11 | 81± 8 | 0.4 |
| Cardiac output (L/min)^‡^ | 8 ± 2 | 8 ± 2 | 0.9 |
| Total peripheral resistance (mmHg/L/min) ^‡^ | 11.4 ± 2.6 | 11.5 ± 2.5 | 0.5 |
|  |  |  |  |
| *Metabolic and Hormone Status* |  |  |  |
| Glucose (mmol/L) | 4.1±0.3 | 5.2±0.9 | **0.0002** |
| Insulin (mmol/L) | 52.1±25.1 | 201.9±164 | **<0.0001** |
| Estradiol (pmol/L) | 59168±18410 | 79648±58424 | 0.2 |
| Progesterone (nmol/L) | 514.2±244.4 | 495±169.1 | 0.9 |
| Testosterone (nmol/L) | 2.3±0.7 | 3.5±1.5 | **0.007** |

^*^Pre-pregnancy BMI was calculated using self-reported pre-pregnancy weight. ^†^ Gestational weight gain category was determined using the Guidelines for weight gain during pregnancy.(1) ^‡^ Cardiac output and total peripheral resistance were calculated using the ModelFlow algorithm.(45) Groups were compared using unpaired t test or Mann-Whitney test when appropriate. Categorical variables were analyzed Fisher’s exact test. Data expressed as mean ± standard deviation.
